# The significance of growth shells in development of symmetry, transparency, and refraction of the human lens

**DOI:** 10.3389/fopht.2024.1434327

**Published:** 2024-07-19

**Authors:** Teri M. Greiling, Judy M. Clark, John I. Clark

**Affiliations:** ^1^ Department of Dermatology, School of Medicine, Oregon Health & Science University, Portland, OR, United States; ^2^ Department of Biological Structure, University of Washington, Seattle, WA, United States; ^3^ Department of Biological Structure & Ophthalmology, School of Medicine, University of Washington, Seattle, WA, United States

**Keywords:** lens development, growth shells, symmetry, transparency, refraction, microcirculation

## Abstract

Human visual function depends on the biological lens, a biconvex optical element formed by coordinated, synchronous generation of growth shells produced from ordered cells at the lens equator, the distal edge of the epithelium. Growth shells are comprised of straight (St) and S-shaped (SSh) lens fibers organized in highly symmetric, sinusoidal pattern which optimizes both the refractile, transparent structure and the unique microcirculation that regulates hydration and nutrition over the lifetime of an individual. The fiber cells are characterized by diversity in composition and age. All fiber cells remain interconnected in their growth shells throughout the life of the adult lens. As an optical element, cellular differentiation is constrained by the physical properties of light and its special development accounts for its characteristic symmetry, gradient of refractive index (GRIN), short range transparent order (SRO), and functional longevity. The complex sinusoidal structure is the basis for the lens microcirculation required for the establishment and maintenance of image formation.

## Introduction

Symmetry, refraction and transparency are optical properties of the biological lens required for image formation in the human eye. Studies of lens growth and development across species report that exponential growth is continuous throughout life without loss or replacement of cells. A typical model for exponential growth is: W = Wm e^–k/A^ (where “W” is dry weight, “Wm” = maximum weight, “k” is rate of growth, and “A” is postnatal age) (1, 2). Lens dimensions increase synchronously and continuously through the addition of symmetric growth shells. These growth shells form a complex sinusoidal structure that forms the basis for the lens microcirculation and the formation and maintenance of transparency.

## Brief summary of lens embryology

### Lens placode

**Figure 1:**
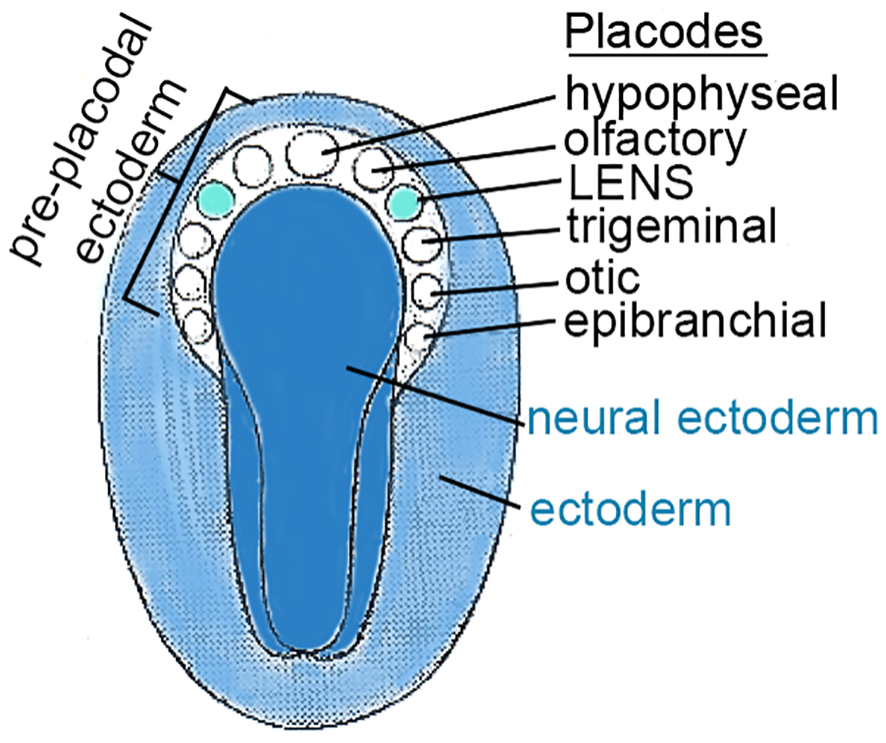
CRANIAL PLACODES in the TRILAMINAR EMBRYO. This figure represents a dorsal view of the ectodermal surface of a trilaminar embryo, prior to neural tube formation (K. Altdorfer, https://slideplayer.com/slide/12872844/). As the neural plate forms, a horseshoe shaped region peripheral to the cranial end of the neural ectoderm, called pre-placodal ectoderm (PPE), forms localized thickenings, “cranial placodes”, for specialized structures including the lens. A number of cranial placodes are indicated by circles and labeled. The lens placode responds to signaling pathways involving FGF, BMP, Notch, Wnt, betacatenin, and others described elsewhere in the text.

**Figure 2:**
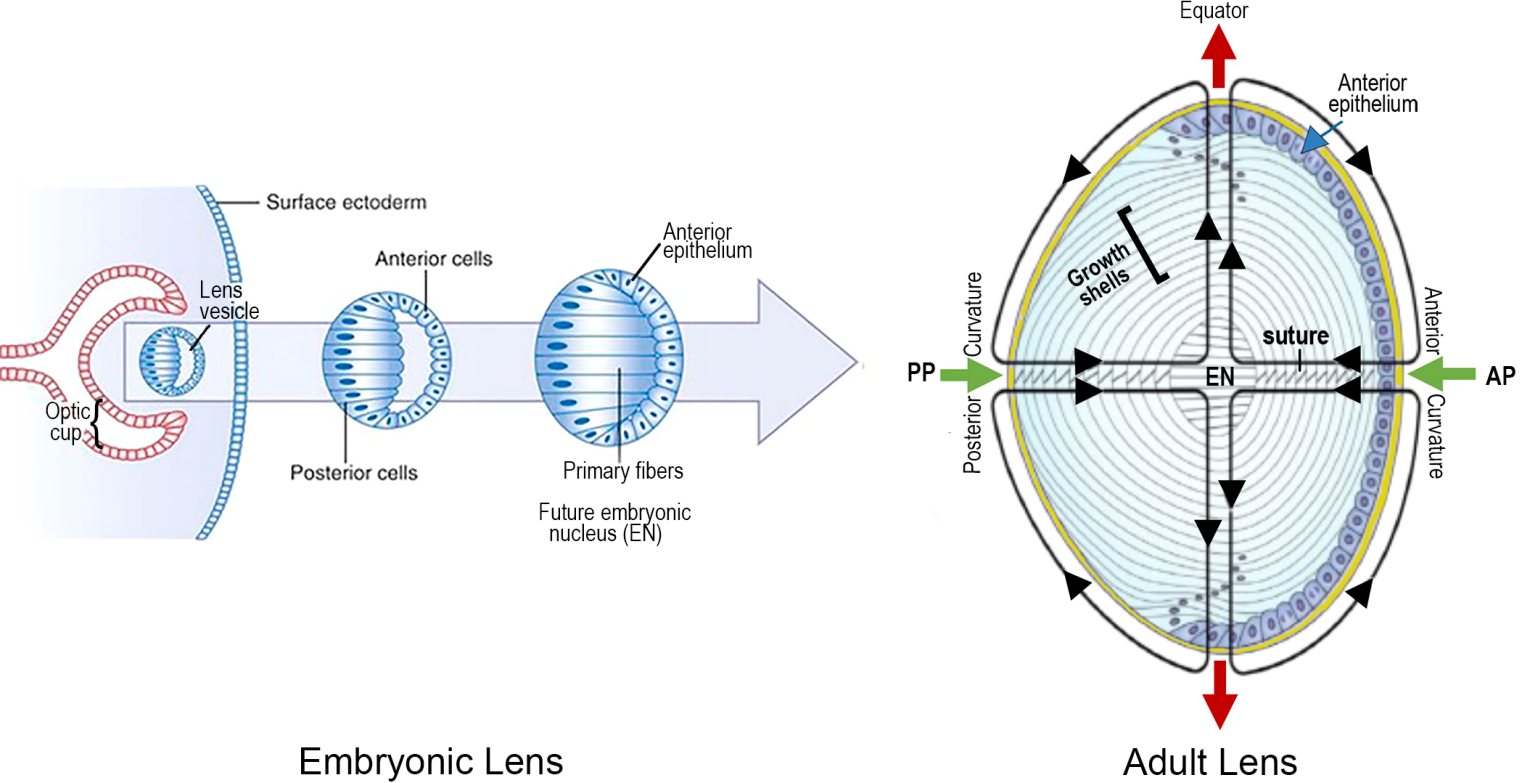
LENS DEVELOPMENT is a MULTISTEP PROCESS BEGINNING at ~3 to 4 WEEKS of EMBRYONIC AGE in the HUMAN: An optic cup extends toward the surface ectoderm from the neural tube, deep to the lens placode. The optic cup induces invagination of the lens placode, followed by its separation from the surface as the lens vesicle. Hyaloid vessels (not shown) supply the developing lens briefly, before regressing. Maturation of the lens vesicle is accompanied by lengthening of the posterior lens cells, adjacent to the optic cup, to form “primary lens fibers”. These cells lose their nuclei and close the vesicle to create a solid cellular mass. In contrast, the anterior cells become an anterior epithelium that maintains its proliferative ability. Synchronous proliferation, migration and elongation of waves of epithelial cells generate “secondary” lens fibers that form growth shells. The growth shells surround the primary fibers of the “embryonic nucleus” (EN), to expand the size of the lens as the optics adjust to the growing eye. The entire lens mass develops within a thick basement membrane capsule (thick yellow line). An adult lens is refractile, transparent and biconvex, consisting of concentric layers of lens fiber growth shells. The functional viability and plasticity of an adult lens is prolonged through a unique microcirculation that nourishes, hydrates and maintains normal electrophysiological homeostasis as the lens grows and adjusts to the optical needs of the growing retina. The image on the right summarizes the microcirculation: Green arrows indicate the inflow of fluid at the anterior (AP) and posterior (PP) poles at the center of the anterior and posterior curvatures. Red arrows represent the fluid outflow at the equator. A number of growth factor pathways are essential for regulation during both embryogenesis and growth shell formation.

At ~3 weeks of gestation in humans, a small number of cells (50 to 100) swell, thicken, and form a lens placode at the edge of the neural plate (neuroectoderm). This lens placode is the origin of the cells that generate the refractile, symmetric, transparent lens (**Figure 1**). The cells in cranial placodes resemble neural progenitors that differentiate into sensory neurons characterized by cytoskeleton-enriched processes, forming dendrites and axons, extending from a cell body containing a nucleus and plentiful organelles to support cell-to-cell connectivity (3–7). In contrast to cells in the sensory placodes, cells in the lens placode swell, thicken and invaginate to form a fluid-filled lens vesicle superficial to the developing optic cup (future retina) (**Figure 2**) (3, 8–11).

### Lens vesicle becomes the lens nucleus

Elongation of the posterior cells in the lens vesicle closest to the optic cup results in a solid cellular mass with the apical surfaces facing inward and the basal surfaces outward. The intercellular space is compressed, and these cells become the “primary” fibers of the “embryonic nucleus”, supplied temporarily by the hyaloid artery, a branch of the ophthalmic artery to the optic cup (2, 3, 11, 12). The anterior cells of the lens vesicle distal to the optic cup are not induced to elongate and remain an epithelial monolayer covering the anterior surface of the embryonic nucleus, and the germinative center for future growth shells (**Figure 2**). (NOTE ABOUT TERMINOLOGY: Early studies suggested the lens was a single giant cell, with a pale yellow nucleus at the core, surrounded by a clear albuminoid cortex, like an egg. When the lens was confirmed to be a cellular tissue, use of the terminology “cortex” and “nucleus” continued to describe cells in the lens periphery and lens center, respectively (13, 14).

### First growth shells

**Figure 3:**
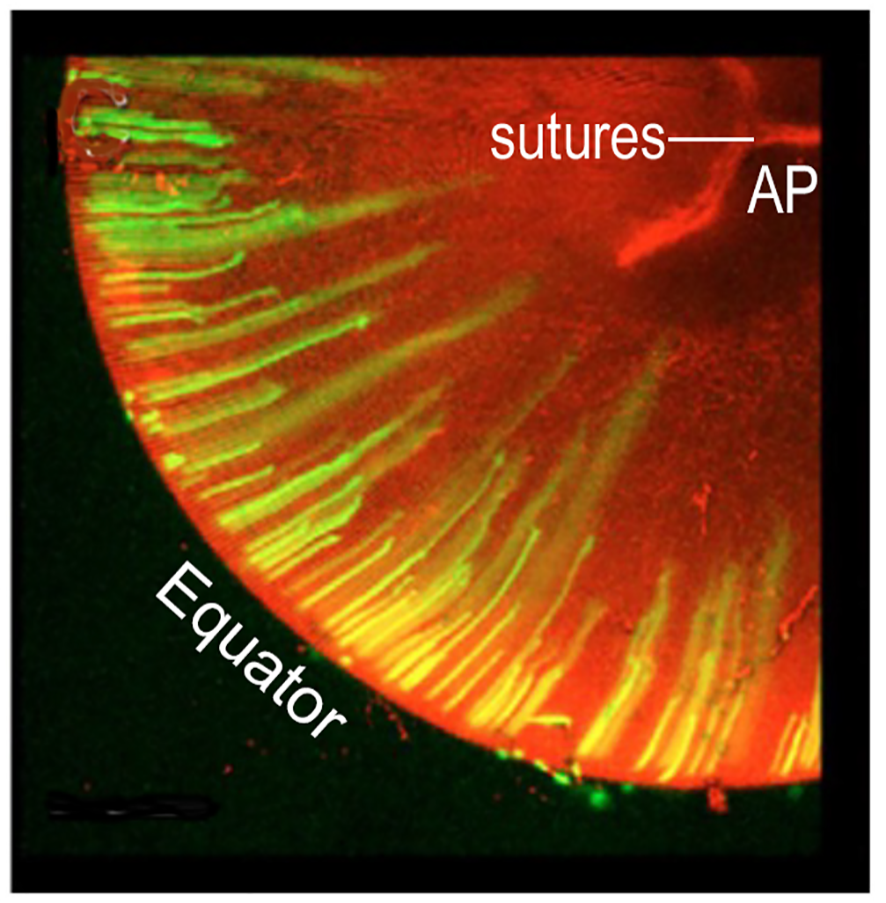
GROWTH SHELL FORMATION BEGINS WITH COORDINATED, PERIODIC ELONGATION of EPITHELIAL CELLS at the EQUATOR. Initially, anterior and posterior elongation of lens epithelial cells produces straight segments that form a discontinuous band around the lens periphery. In this image, green fluorescent protein, GFP, labels the cells elongating away from the equator. With continued elongation, a small number of these fibers will attach at the anterior (AP) or posterior (PP) poles separated radially by ~120 deg, (2.09 radians) (not shown). These are the straight (St) fibers that orient all other cells to fill in the growth shell. The optic axis (not shown) connects the AP with the PP. Elongation is synchronous and coordinated. In the figure, the longer fibers are believed to be forming one growth shell and the shorter fibers are beginning to elongate to form the next growth shell. Growth shells form concentric layers observed in intact lenses. (modified from Shi et al. (2009) J. Cell Sci. 122:1607-15).

The cells in the anterior epithelial layer can proliferate and migrate toward the equator of the developing lens, where they organize into ordered meridional rows. Synchronized elongation of the meridional cells, posteriorly and anteriorly, initiates the formation of a coordinated band of arc-shaped secondary cells, parallel to the optic axis. This band will become a growth shell at the peripheral lens cortex (**Figure 3**) (2, 9, 11, 15, 16). As the temporary vessels of the hyaloid vasculature regress, each growth shell becomes the developmental mechanism for adding symmetric layers of new “secondary” fibers that increase the size of the lens during formation of the visual system (**Figure 2**). New growth shells contain malleable, refractile, organelle-free secondary fibers that subsume previous shells surrounding the lens nucleus (17–20). New growth shells can adjust to the optical needs of a growing eye (16, 21).

**Figure 4:**
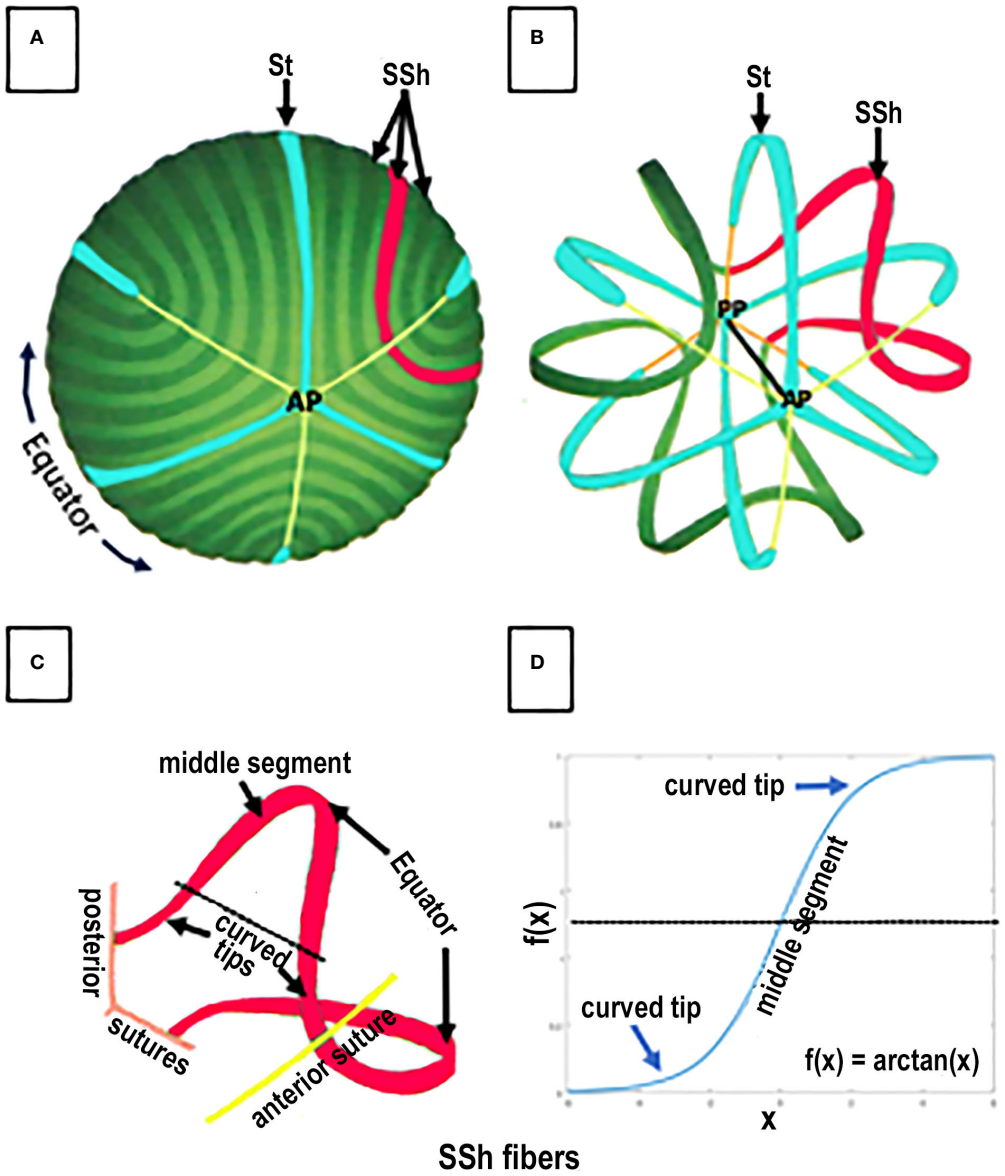
SPACE FILLING and FIBER ORGANIZATION in a GROWTH SHELL. An anterior view of the lens fibers in a typical GROWTH SHELL is shown in **(A)**. The dark and light green bands, each representing 20 individual fibers, are modeled from scanning electron micrographs of the growth shell surface. A few straight (St) fibers (turquoise) attaching at the anterior pole **(B)** are organizing centers for the many S-shaped (SSh) fibers (example highlighted in red) that fill-in the developing growth shell forming the anterior and posterior curvatures of each hemisphere in the biconvex lens **(A, B)**. Three sutures originating at the anterior (AP) poles are shown as thin yellow lines. Removing a number of fibers (in the figure) allows both the anterior (AP) and posterior (PP) poles to be seen **(B)** where three attachments for St fibers and three sutures (orange line) are shown. The optic axis (black line) connects the anterior and posterior poles. Space filling is a complex process of fiber assembly. During the start of fiber elongation, the middle segment of each SSh fiber is parallel to the optic axis (as in **Figure 3**). While one tip of an St fiber (blue) attaches at either the anterior (AP) or posterior (PP) pole, the other tip is the origin of the sutures **(A, B)** and the St fibers remain parallel to the optic axis. In contrast, the tips of elongating SSh fibers (red) do NOT attach at either pole. Instead, the elongating tips of SSh fibers curve away from the poles, toward the plane of the orienting St fibers, where they join the curved tips from a corresponding SSh fiber, to form a pair and create a suture (**B** red). For example, **(C)** represents a pair of red SSh fibers. An anterior tip curves to meet the curved tip of an SSh fiber (red) elongating from the opposite side of the St fiber and form anterior (yellow) suture. Note that the SSh fibers are not attached at a pole and each new connection between the SSh fibers lengthens the suture. Similarly, the posterior tip of the same red SSh fiber curves to meet the tip of a corresponding SSh fiber (not shown) to form and lengthen the posterior suture **(C)**. Both sutures are shown in the figure, but the suture is only formed when the tips of each SSh pair connect. In this way pairs of elongating SSh fibers fill in a GROWTH SHELL as they establish the sutures. (NOTE about TERMINOLOGY: The SSh fibers are described as having “opposite end curvature” by Kuszak because they curve away from the St fibers and the poles. In a 2-D projection of an SSh fiber **(D)**, each SSh fiber can be described as having a distinctive symmetric, sigmoid shape with the two curved tips extending away from a straight middle segment **(D)**. The sigmoid function is defined as f(x) = arctan(x). It is well established, but not widely appreciated, that when SSh fibers meet other SSh fibers, the pairs of SSh fibers fill in and form the posterior and the anterior surface of a GROWTH SHELL. When SSh pairs connect, the sutures are formed. Altered sutures are an indication of abnormal lens fiber differentiation and function.

It is important to emphasize that the coordinated addition of symmetric growth shells of secondary fibers expands the size of the lens and creates a biconvex, biological spheroid that functions as an optical element in the human eye (**Figure 4**) (11, 19, 22–24). The spheroid is defined by an equator separating the anterior from the posterior hemisphere. The radius of curvature of the surface anterior to the lens equator is ~10mm and the radius of curvature of the surface posterior to the equator is ~6mm (see **Figure 2**) (25, 26). With the growth of the visual system, the optical curvatures vary slightly as they adjust the focal length of the lens to the dimensions of the changing eye (16, 27). Throughout development of visual function, the optics of the growing lens are carefully synchronized with the establishment and maintenance of optical quality during the life of an individual (16, 22, 28). As a mechanism for the development of optics, growth shells are an unprecedented success.

### Cellular specialization

In the absence of blood vessels, each growth shell develops symmetric layers of cellular fibers, containing condensed cytoplasmic proteins, largely crystallins and cytoskeleton, to increase the refractive index, and establish transparent short-range order necessary for focusing images on the retina (3, 22, 29). Growth shells can do more. Without blood vessels, structural specializations in the fibers of growth shells contribute to a symmetric circulatory system for fluid flow that regulates hydration, ionic homeostasis, and uniform distribution of nutrients, in support of dynamic growth to optimize the optics for the growing, changing visual system (**Figure 2**) (3, 7, 12, 30, 31). Without a functioning microcirculation, the lens cannot develop the symmetric gradient of refractive index (GRIN) and transparency required for image formation in the growing visual system (32–35). In fact, the lens might as well be a piece of glass or plastic. Instead, nature created a growth shell mechanism for the biological lens, that is unique in all of developmental biology (36, 37).

## Growth shells

### Structure: straight and S-shaped fibers

Each growth shell is comprised of two types of secondary fibers: straight (St) and S-shaped (SSh) fibers (**Figure 4**) (22, 36). St fibers are crescent-shaped, parallel to the visual axis, and attached to either the posterior or anterior pole (**Figures 4A, B**), where they become growth centers for the anterior or posterior hemispheres of the growth shell. Posterior to the lens equator, St fibers radiate away from the posterior pole, separated by 120 degrees (**Figure 4B**) and anterior to the lens equator, St fibers radiate away from the anterior pole separated by 120 degrees (**Figure 4B**). Notice that the tips of the elongating St fibers stop short of the opposite poles, ending at the tips of the Y suture (**Figure 4B**) (15, 22, 38).

The second type of lens fibers, the SSh fibers, fill in the growth shell (**Figure 4A**). SSh fibers are oriented along, and adjacent to, the St fibers (**Figures 4A, B**). SSh fibers have three parts: a straight middle segment, parallel to the St fiber, and two tips, curving away from the St fiber, to meet curved tips from other SSh fibers forming a pair of SSh fibers (**Figure 4**, red fibers). Anterior to the equator, where the curved tips meet, the anterior suture forms, and posterior to the equator, where the curved tips meet, the posterior suture forms. The sutures are formed where pairs of SSh fibers meet, anteriorly or posteriorly (**Figure 4**, red fibers). Because the tips curve to meet other SSh fibers, they appear as symmetric, sigmoid-shaped fiber cells when projected in 2-D (**Figure 4D**). The trigonometric function describing the SSh fiber is f(x) = arctan(x), which is the basis for the symmetry of a growth shell and accounts for the symmetric index of refraction, transparency, and the anterior and posterior curvatures of the lens.

### Structure: sinusoidal networks form Y sutures

Where the curved tips meet anteriorly, the sutures are positioned in a “Y-shape” and posteriorly, the sutures form an “inverted Y-shape” (22). The anterior and posterior sutures are not aligned and are offset by 60 degrees. This is because of the sigmoid shape of SSh fibers (**Figure 4D**). Because the SSh tips curve in opposite directions away from their middle (**Figure 4C**), they are no longer in the same anterior/posterior plane (22, 37–39). Still, symmetry is maintained in normal development. The result is a continuous, interconnected sinusoidal network of fibers throughout the entire growth shell. Throughout the growth shell, SSh fibers in the posterior hemisphere connect directly with the fibers in the anterior hemisphere. It is easy to understand how reactive oxygen species, advanced glycation end products, inflammatory agents, osmolytes, or other systemic stresses can disrupt the coordinated, symmetric elongation of fibers and alter the suture patterns.

**Figure 5:**
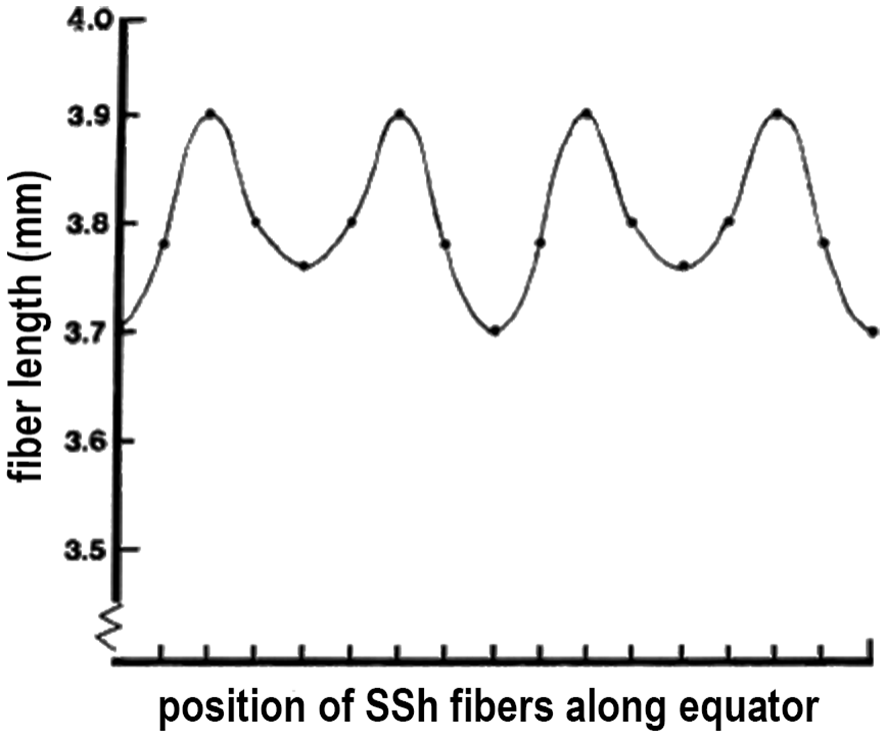
VARIATIONS in FIBER LENGTH in a GROWTH SHELL. Because the pairs of elongating SSh fibers intersect the equator, the length from the equator to fiber tip is expected to be unequal. When actually measured and plotted, an unexpected finding is that the variations in length are regular and periodic. Periodic oscillations in lens fiber lengths represent a sinusoidal pattern, reflecting the careful synchronization in growth shell formation.

Careful analysis of electron micrographs of developing lenses confirms that sutures in a growth shell are formed by the connections between differentiating SSh fibers (22, 36, 38–40). In contrast, the embryonic lens nucleus at the center of the adult lens has no sutures and consists of the primary fibers that elongated to obliterate the lens vesicle and establish the original cell mass (**Figure 2**). Subsequently the embryonic nucleus is overlain by secondary fibers (22, 38). As lens development continues, secondary fibers organize into isomorphic interconnected growth shells in a coordinated, synchronized process. The amount of curvature in the individual tips of the SSh fibers varies relative to position relative to where the pairs meet and establish a suture (**Figure 4**) (39). An unexpected result is the normal variability in the lengths of the SSh fibers connecting along the suture lines. The lengths oscillate with a regular, sinusoidal pattern, another indication of coordinated, synchronization of growth shell formation (**Figure 5**). It should be noted that the ends of secondary fibers expand and overlap at the sutures (39, 40). The overlap is part of the 3-D interconnected suture planes, extending from the surface into the embryonic nucleus. The suture can act as a channel carrying fluid containing ions, nutrients, soluble factors, and antioxidants that regulate and maintain symmetric structure (7, 22, 41, 42). When normal fiber differentiation is disrupted, the sutures appear abnormal (22).

### Structure modeling

**Figure 6:**
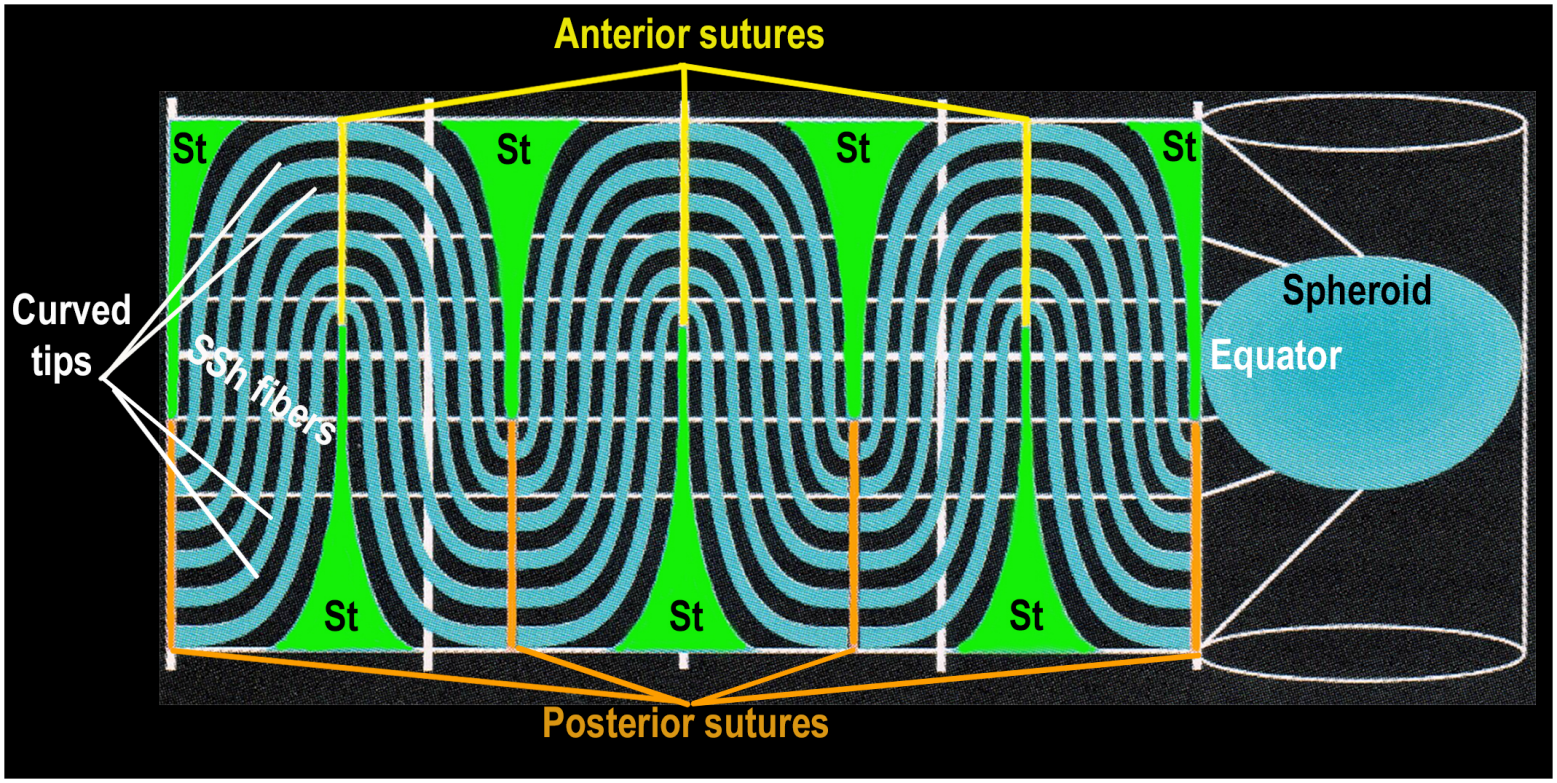
SYMMETRY is DETERMINED by TWO TYPES of DIFFERENTIATED FIBER CELLS. This is a 2-dimensional projection of a GROWTH SHELL. The computer aided drawing software generates a 2-D map by projecting the 3-D lens spheroid (growth shell) onto a 2-D cylindrical surface (**Figure 6** right side). It is known as a projection because it simulates a bright light placed inside the spheroid so that any point (x,y,z) on the surface of the 3-D spheroid is projected to a point (x,y) on the surface of a cylindrical screen, surrounding the spheroid (**Figure 6**, right side). The lens equator is represented as a horizontal white line on the cylinder, between the anterior and posterior poles, represented as the open ends of the cylinder. When the computer “unfolds” the cylinder, the most obvious structural features of the 2-D projection of a typical growth shell are the symmetric oscillations of the SSh fibers (black and turquoise) above and below the equator (dashed white line), centered on the St fibers (green), positioned ~60 deg (1.05 radians) apart. The St fibers appear “cone shaped” with a wide base near the pole and a narrow tip at the beginning of each suture, near the equator. This distortion of St fiber dimensions is the result of an increase in scale near top and bottom of the cylinder, making the St fibers appear disproportionately large at the poles. (The St fibers are represented accurately in **Figures 4A, B** where their relative size is the same size as that observed *in situ* in electron micrographs of actual lenses.) In the 2-D map, the anterior sutures are represented by yellow vertical lines at the top of the 2-D map and the posterior sutures are orange vertical lines at the bottom of the 2-D map. Even though distorted, the St fibers extend from a pole to a tip of each suture near the equator (white line). The curved S-shaped (SSh) fibers form the oscillating pattern filling the growth shell. One curved tip of the SSh fibers connects to a posterior suture and the other curved tip connects to an anterior suture. The extensive fiber interconnections throughout the growth shell form a symmetric sinusoidal pattern essential for the organization of a symmetric microcirculation. This fiber symmetry is the basis for establishment and maintenance of refraction and transparency in an effective optical element in the human lens.

When first observed, the unusual organization of St and SSh fibers is a bit confusing and difficult to understand. To visualize the details and the overall structure of a growth shell during development, growth, and aging, Kuszak chose 2-D projections (22, 36–38, 40). Using computer aided drawings, he applied geometric methods known since Babylonian times for navigation of the earth (a spheroid) to study fiber patterns in growth shells of the biological lens (also a spheroid) (**Figure 6**) (22, 38). Combining computer aided drawing with thorough, careful scanning electron microscopy, Kuszak revealed new information about the assembly of symmetric growth shells in the lens. His results represent the synchronous differentiation that is the structural basis for both the lens optics and the symmetric microcirculation of fluid throughout the lens, in the absence of vasculature. When differentiating fibers in the growth shells are exposed to fluid influx through posterior and anterior sutures, they can respond to the fluid contents like ions, nutrients, and soluble factors controlling lens growth. The extensive connectivity between SSh fibers in the growth shells, specifically posterior and anterior to the equator, accounts for the symmetry of the posterior and anterior curvatures of the developing lens. The importance of two organizing centers at the posterior and anterior poles is very clear. While growth shells were recognized previously in lens research, the 3-D computer aided drawing provides much greater detail about their symmetric structure and, potentially, their functional significance. The collective interactions between component St and SSh fibers account for the connectivity and symmetry in the growth shells (22, 36, 37).

### Function in microcirculation

Growth shell formation is not simply a space-filling exercise. It is the basis for a highly connected, complex cellular network, organized to provide maximal image quality to millions of photoreceptors in the visual system. Lens growth is carefully regulated to form a symmetric, refractive optical element that transmits light waves for the formation of accurate visual images (7, 31). It is often unappreciated that a lens consists of diverse populations of fibers constituted from various protein and membrane specializations completed at very different ages across the life of the organism. The process of growth shell formation is the foundation for the symmetry necessary to optimize optical quality, including the gradient of refractive index, GRIN, and transparency in a growing eye.

Many biologists accept the correlation between structure and function (11, 37, 40, 43, 44). As explained in the legend of **Figure 6**, the 2-D map of the growth shell introduces distortion at the anterior and posterior poles enlarging the dimension of the St fibers (**Figure 6**). Regardless of the distortion in a 2-D map, the regular, symmetric pattern of oscillating fibers posterior and anterior to the lens equator is expected to be critical for the growth shell mechanism of lens development (**Figure 6**) (22). The vertical clefts labeled “St” are the straight (St) fibers. Together with the sutures, St fibers are positioned every 60 degrees (1.05 radians) forming regular orienting centers for “waves” of SSh fibers (wavelength 120 degrees or 2.1 radians) above and below the equator (**Figure 6**). The periodic oscillations are oriented to the positions of the St fibers and sutures anteriorly and posteriorly. In the absence of vasculature, a simple hypothesis is that these sinusoidal oscillations are the structural basis for uniform symmetric fluid flow, known as microcirculation. If St fibers are spatial organizing centers for symmetric SSh fiber elongation, then the microcirculation can facilitate uniform fluid flow into the growth shells, anteriorly and posteriorly, to carry nutrients, growth factors, and protective molecules deep into the lens to organize and maintain function (**Figure 2**) (7, 42, 45). Recent studies report the importance of uniform, symmetric fluid flow in the control of hydration, ion homeostasis, refraction, and transparency in the biological lens (31, 32, 42, 45–47).

Few examples of symmetry in a biological tissue are more impressive than the experimental measurement of the loops of current flow in a biological lens (**Figure 2**) (46, 48–53). At the time it was reported, the significance of the symmetric current inflow and outflow in lens fiber symmetry and function was unrecognized (48, 53). Now, penetration of nutrients, metabolites, ions, and soluble factors is thought to occur through influx of fluid into the anterior and posterior suture planes, established by the alignment of sutures during the synchronized formation of growth shells (**Figure 6**). Fluid efflux occurs at the equator through an intercellular outflow pathway thought to be mediated by gap junctions. Hydrostatic pressure as high as 335 mm Hg centrally, falls to 0 mm Hg at the periphery, creating a pressure gradient for driving flow. The activity and localization of channels in the fiber membranes regulate flow and are critical for the optics of the visual system (7, 11, 31, 42, 45, 47, 50, 54).

There is much to learn from the lens about biological symmetry. The growth shell mechanism is a rare example of a developmental process for continuous formation and maintenance, year after year, of a highly symmetric refractile, transparent tissue, and the establishment of corresponding microcirculation. No other cellular tissue in the human compares with the transparent lens for studies of complex molecular and cellular function over a lifetime. Lens structure and function for image formation is intimately linked to symmetry, the gradient of refractive index (GRIN), and transparent short range order. In the eye, the dynamics of collective, often complex, interactions, at the molecular and cellular levels are accessible to modern, non-invasive methods of research in living individuals.

The current hypothesis that the microcirculation is a primary factor in the formation of the biconvex, human lens, places an emphasis directly on the significance of fiber membranes (45, 55–58). The 1000-fold elongation of the SSh secondary lens fibers is achieved through a dramatic expansion of the membrane surface area (22, 38, 59–63) accompanied by an elaborate reorganization of the lens fiber cytoskeleton (11, 60, 61, 64–69). It is well established that lens fiber membranes are specialized to facilitate fluid flow throughout the decreased intercellular spaces. During the formation of a growth shell the cytoskeleton condenses at the periphery of the hexagonal fibers, as a dramatic increase in the proportion of membrane cholesterol accompanies the increase in fiber membranes, and the intercellular spaces are narrowed (11, 70–75). Intuitively, a decrease in the extracellular space might be expected to increase resistance to fluid flow through the lens microcirculation. Studies of microfluidity suggest the opposite effect (76–79). High cholesterol can stabilize membranes, increase hydrophobicity to decrease surface tension, and help move fluids through the microcirculation of the lens (80). The complexity and heterogeneity of fiber membranes and their microenvironment make the study of microcirculation a challenge. While discussion of the origins of the microcirculation in the growth shell mechanism is beyond the scope of this article, growth shells are incredibly important as a foundation for development of the lens as an optical element in the human eye (58).

When the space between cell membranes decreases, the resistance to turbulent flow can decrease to favor laminar flow, increasing microfluidity (77, 79). The microfluidity between membranes can be enhanced further by an increase in the area of hydrophobic surface, reducing interactions between aqueous fluid and charged membrane phospholipids (80). Increased membrane cholesterol resists oxygen permeability favoring elimination of intracellular organelles (81, 82) and stabilizes the elongated fiber shape, the condensation of the cytoskeleton at the cell periphery, the establishment of transparent short range order, phospholipid surface projections, and a decrease the intercellular spaces (83–85). A dynamic cytoskeleton compresses and stabilizes the cell membrane and positions membrane channels, cell adhesion molecules, and connexins along the cell surface. The constructive effects of high cholesterol levels in fiber membrane can contribute to lens microcirculation and improve symmetry, transparency, and GRIN in the lens as new symmetric growth shells are added (82, 84–86).

When growth shells are added at the lens periphery, they seem most responsive to constituents of the microcirculation. There appears to be a narrow band of growth shells forming a supranuclear region between the lens nucleus and cortex, where plasticity permits fiber reorganization (17, 87–90). Both electrophysiology and light scattering results indicate a subtle change several layers deep to the surface, consistent with an electrophysiological syncytium, and/or a network of interacting proteins and membranes (16, 17, 27, 90–92). Apparently, supranuclear fibers in new shells share plasticity to remodel the surface curvatures and adjust the biconvex lens to changes in optical requirements as the eye grows. The plasticity that accounts for variations in light scattering appears be sensitive to intracellular modifications associated with clinical conditions specific to light scattering phenotypes, including myotonic dystrophy, genetic mutations, Down Syndrome and Alzheimer’s Disease (89, 93–95).

### Plasticity of growth shells

Plasticity of the lens growth shells allows for subtle improvements in the optical properties as eyes grow from youth to adult (16, 96, 97). Developmentally, dramatic plasticity is demonstrated in lens inversion experiments (98, 99). After removal from the optic cup, a developing lens can be rotated 180 degrees, and then replaced in the eye so that the epithelium now faces the vitreous (posteriorly) instead of the aqueous (anteriorly). After replacement, repolarization occurs so that the (now) posterior epithelium elongates to fill-in the lens vesicle and a “cap” of new epithelium forms anteriorly facing the cornea. The results represent an extraordinary malleability that allows the newest growth shells to respond to factors carried through the microcirculation from the anterior aqueous and/or posterior vitreous. In a separate example of plasticity, a second lens mass develops in a mutant zebrafish, apparently because of two growth centers (100). One of the most extreme examples of lens plasticity during normal development is the lens of the “four-eyed” fish, Anablebs anablebs (101–103). The two growth centers in the growth shells account for the formation of a pyriform-shaped lens that focuses light waves simultaneously, from two separate environments: air and water, onto separate regions of the same retina. In a 2-D map of a growth shell, individual SSh fibers are exposed to both the posterior and anterior environments by an influx of fluid carried through their posterior or anterior sutures (**Figure 6**). Lens fiber plasticity permits the posterior and/or anterior curvatures to adjust refraction in response to either environment (posterior or anterior).

Each growth shell generates symmetric posterior and anterior convexities, with different radii of curvature, to adjust lens optics for precise focusing of images on a growing, expanding retina with minimal spherical aberration. Generation of two biconvex surfaces in the biological lens is achieved when posterior and anterior growth centers are established by the St fibers. Orientation of the SSh fibers, connecting at the sutures posteriorly and anteriorly, forms two biconvex surfaces that optimize the optics of the visual system. The number and orientation of lens fibers need to adjust the size of each new growth shell with age, to conform to the principles of image formation in the changing human eye (19, 22, 104).

### Regulation of growth shell formation

Growth shell development is regulated largely by growth factor and signaling pathways involving FGF, BMP, IGF, TGFbeta, Notch, wnt, PDGF, and others (2, 3, 10, 23, 57, 105–112). Numerous studies support the hypothesis that concentration gradients of FGF and BMP are central to the regulation of elongation and maturation of lens fibers (14, 113). These are reviewed in detail elsewhere (10, 14). Both the levels of the growth factors in aqueous and vitreous and the locations of their receptors in the lens regulate formation of the symmetric growth shells (108, 111, 114, 115). The impact of these regulatory pathways on lens growth and differentiation is so important that there is systematic redundancy, so that IGF, EGF, TGFbeta, and other soluble factors contribute to formation of a growth shell. Redundancy benefits and protects the effectiveness of the growth shell mechanism in the formation of symmetric, concentric spherical layers. Given the complexities of the relationships between growth factors, signaling pathways and, gene regulatory networks on fiber differentiation, the importance of the synchronization of growth shell formation is sometimes overlooked. Bursts of transcription are a direct measure of the synchronized fiber differentiation in the coordinated development of the growth shells, and are necessary for generation of symmetry (10, 32, 116–118). The pulsatile activity of PDGF and the discovery of PDGF receptor in distal regions of lens epithelium where synchronicity is initiated, altered our understanding of the regulation of periodic symmetry in the biological lens (106, 119–123). Correlation of the cellular distributions of growth factors and receptors will clarify the link between growth factor activity and coordination of the remarkable geometric patterns (**Figure 6**) accounting for symmetry, GRIN, and transparent short range order in the biological lens.

Research continues to demonstrate the importance of synchronization of growth factors in regulating development and maintenance of symmetry, GRIN, and transparency in the biological lens (2, 10, 105, 106, 109–113, 119–122, 124–126). Differentiation of symmetric, concentric layers of elongated, denucleated, transparent, refractile fiber cells in the lens spheroid is complex and represents unprecedented spatio-temporal regulation in biology (11, 22, 37, 38, 121). Although the lens is ordered at all scales of structure, from molecules to the whole tissue, the “crystalline” biological lens is not crystalline. It is formed by concentric shells of symmetric lens fibers (**Figures 2**, **4**, **6**). Complex, synergistic, and cooperative, often overlapping, signaling pathways promote structural and functional longevity of lens function (11, 24) Current research needs to consider interactions within networks of growth factors in the regular and coordinated assembly of new growth shells in the mechanism of lens development.

## Discussion

It is important to realize that the radius of curvature of a growth shell is symmetric both anteriorly and posteriorly. The precise dimensions are carefully regulated to maximize the optical function of the biconvex, biological lens. The St fibers attached at the posterior and anterior poles form two spatial organizing centers, separated by the equator, in each growth shell (**Figures 4**, **6**). Posterior to the lens equator, the St fibers orient the SSh fibers to form a posterior convexity, and anterior to the equator the St fibers orient SSh fibers to form an anterior convexity. The focal point of a biconvex lens depends on the symmetric gradient in the index of refraction, and the curvature of both the anterior and posterior surfaces. While formed by secondary fibers in the peripheral cortex, each GROWTH SHELL is dynamic and plastic. The surface curvatures of the posterior and anterior hemispheres can adjust to the changing optical requirements of image formation during development and growth of the eye (97, 127, 128). As the lens grows, the newest growth shells (**Figures 3**, **4**) are added between the elongating cortical fibers and the deeper established growth shells. Between the deep cortex and the superficial nucleus, new growth shells form a thin layer that can respond and continuously modify the optics of the growing eye. In contrast, fibers in older, deeper growth shells become highly interconnected and stabilized in the nucleus, in what is known as an electrophysiological “syncytium” (17).

**Figure 7:**
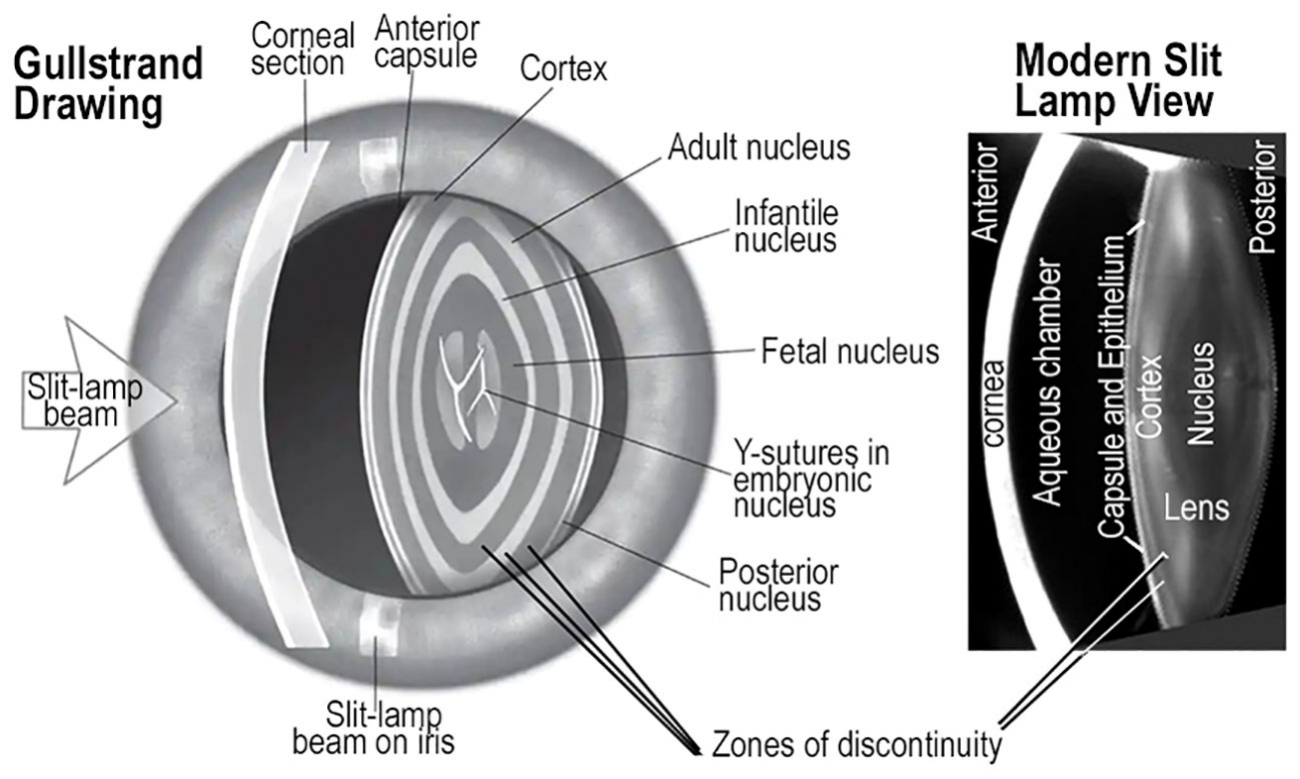
ZONES of DISCONTINUITY and GROWTH SHELLS. Drawings made by Gullstrand of the human eye resemble a photograph from a modern slit lamp. Both show oscillations in light scattering known as zones of discontinuity. In both images, the variations in light scattering appear as concentric, symmetric layers in the lens, and are consistent with the growth shell mechanism of lens development and aging. The Gullstrand drawing appears to have much more prominent scattering from the zones of discontinuity than the modern photo image. The main point is that zones of discontinuity in the refractile, transparent lens of living patients are concentric and symmetric, consistent with growth shell structure. These images confirm the value of direct analysis of lens structure and function in living individuals across a broad range of ages using modern optical technology.

It should be emphasized that not all light scattering is the same. Light scattering depends on wavelength, intensity and scattering angle, the index of refraction, the size, shape and concentration of scatterers, their interactions, symmetry, order, and other biophysical parameters including pressure, temperature, and concentration (129–133). The diversity of differentiating cells and fibers generated in growth shells can be evaluated *in vivo* as variations in fiber structure optimize image formation in the human visual system (130, 134–136). The diversity of fibers and the plasticity of growth shells seem to account for the variability observed in the zones of discontinuity (**Figure 7**) (11, 137, 138). Plasticity can account for sensitivity of differentiating fibers to environmental factors including glucose levels, toxic substances, or osmolytes penetrating the lens through the microcirculation to reach the differentiating lens fibers.

When the layered structure of the lens was first observed *in vivo* by Gullstrand, the inventor of the slit lamp, variations in scattered light established the basis for understanding the concentric growth shells. Their symmetry, refraction, and transparency could be observed directly in the human lens (**Figure 7**) (139). Small fluctuations in refractive index gradient and non-random scattering are associated with normal growth and development resulting from modified apoptosis, mitophagy, or autophagocytosis (136, 140–142). These mechanisms are commonly associated with cell death and cell replacement, not prolongation of molecular and cellular longevity, typical of the biological lens (143, 144). In a healthy eye, small fluctuations in the refractive index that produce light scattering from zones of discontinuity, do not impair vision (145) (**Figure 7**). Detailed computer aided drawings of differentiating lens fibers in normal growth shells can explain images commonly recorded in slit lamp examinations (22, 36–38, 41, 65). In a growth shell, decreasing oxidative metabolism results in the loss of organelles and the reduction in reactive oxygen species to improve transparency during growth of the lens. In fact, all primary and secondary fibers formed in a lens are retained for a lifetime. Lens fiber differentiation involves unique protective mechanisms including antioxidants, microcirculation, cytoskeletal stability, post-translational modification (PTM), and high levels of small heat shock proteins (sHSP) to enhance optical function (symmetry, GRIN, and transparency) of a lens (11). Failure to preserve the viability of any fiber is presumed to lead to pathology. Typical lens fiber differentiation, occurring during lens growth, seeks to decrease the dimensions of irregularities in the refractive index, “n”, well below micron sizes. The subtle light scattering from tiny, often temporal, spatial fluctuations in “n”, is known as Rayleigh scattering. These tiny fluctuations in “n” are not readily observed histologically, even in electron micrographs (130, 133, 146, 147). While changes in Rayleigh scattering can be a measure of differentiation of normal transparent subcellular structure in living animals, it can also be predictive of the progressive loss of transparency under unfavorable conditions of molecular and cellular aging (11, 91, 92, 130–132, 148, 149).

## Conclusion

The human lens is not glass, but could be, if nature chose a developmental mechanism different than growth shells. Other ectodermal derivatives in the integument are dehydrated, including hair, nails, feathers, and claws. Dehydration of the fibers of the developed lens is all that is needed to produce a solid, glass-like lens, similar to a camera lens. For example, when a lens is removed from an eye (rodent, cat, dog, zebrafish, other) and allowed to dehydrate slowly, in a controlled laboratory environment, a biological lens can transition into a transparent, refractile solid. Instead, nature chose to (bio)engineer a highly symmetric and interconnected growth shell system of lens fibers, supported by an unusual microcirculation that limits oxidative metabolism and conserves hydration, physiological homeostasis, and uniform nourishment in a cellular lens. In a biological lens, optical function is prolonged in the visual system over a lifetime (7, 30, 31, 47, 56). A relevant comparison can be made between hydration in a lens and the tardigrade, an extremotolerant organism known to be able to maintain its cellular structure under conditions of complete dehydration (anhydrobiosis) (150). Similarly, the fiber structure of a lens must be maintained under conditions of severe dehydration. While there are advantages to conducting research on biological lenses from a materials science perspective, human vision demands more than a piece of glass or plastic. The symmetry of the growth shells not only prolongs the functional life of a lens, it supports dynamic modifications that optimize the optics of the visual system. The growth shell microcirculation is a major physiological innovation. The vascular system in non-lens tissue consists of lymphatics and blood vessels that supply oxygen and nutrients to cells. The vasculature modulates cell and tissue fluid homeostasis. In a lens, oxygen is toxic and lymphatics carry immune cells that can recognize modified, aging cells, like the lens fibers, as abnormal and destroy them. In growth shells, the microcirculation is a natural alternative to the typical systemic vasculature. The growth shell microcirculation regulates hydration and provides nutrition in a protective environment of antioxidants and stress response proteins, to optimize cellular, molecular, and functional longevity of refractile, transparent lens fibers.

Coordinated, synchronous differentiation of lens fibers in growth shells is necessary for the optics of the human eye to adjust as the visual system grows and ages. Even though multiple levels of protection (including post-translation modifications, anti-oxidants, and small heat shock proteins) prolong the biological lens for an unusually long functional life, tiny failures at the molecular level and multifactorial, submicroscopic events can slowly and progressively accumulate and disrupt symmetry and order until a “tipping” point is reached (11, 151–154). The greatest risk factor for loss of transparency is aging of molecular and cellular constituents (11, 24). Membrane specializations (projections, protrusions and connections) between fiber cells change with normal lens development and are associated with formation of the symmetric growth shells. Specific surface features characterizing the boundary of the organelle free zone (OFZ), are not well defined in a normal lens. In abnormal lenses, where fiber differentiation is disrupted, the symmetric relationship(s) between straight and S-shaped fibers in growth shells is distorted, and result in an asymmetric pattern of sutures. (22,37,38). Advances in imaging and analytical sciences suggest that novel integrated research on lens symmetry, GRIN, and transparency in growth shells, will improve our knowledge of natural protection for the optics of individuals at risk for lens opacification that accounts for more than 50% of vision impairment globally (58, 130, 133, 155–157).

## Author contributions

TG: Conceptualization, Data curation, Formal analysis, Validation, Visualization, Writing – review & editing. JC: Conceptualization, Formal analysis, Validation, Visualization, Writing – review & editing, Software, Supervision, Writing – original draft. JC: Conceptualization, Formal analysis, Software, Supervision, Validation, Visualization, Writing – original draft, Writing – review & editing, Data curation, Funding acquisition, Investigation, Methodology, Project administration, Resources.
